# A targeted decision aid for the elderly to decide whether to undergo colorectal cancer screening: development and results of an uncontrolled trial

**DOI:** 10.1186/1472-6947-10-54

**Published:** 2010-09-17

**Authors:** Carmen L Lewis, Carol E Golin, Chris DeLeon, Jennifer M Griffith, Jena Ivey, Lyndal Trevena, Michael Pignone

**Affiliations:** 1 Department of Medicine, University of North Carolina at Chapel Hill - Chapel Hill, NC, USA; 2 Cecil G Sheps Center for Health Services Research, University of North Carolina at Chapel Hill - Chapel Hill, NC, USA; 3 School of Rural Public Health, Texas A&M Health Science Center, College Station, TX, USA; 4 School of Pharmacy, University of North Carolina at Chapel Hill - Chapel Hill, NC, USA; 5 School of Public Health, University of Sydney - New South Wales, Australia

## Abstract

**Background:**

Competing causes of mortality in the elderly decrease the potential net benefit from colorectal cancer screening and increase the likelihood of potential harms. Individualized decision making has been recommended, so that the elderly can decide whether or not to undergo colorectal cancer (CRC) screening. The objective is to develop and test a decision aid designed to promote individualized colorectal cancer screening decision making for adults age 75 and over.

**Methods:**

We used formative research and cognitive testing to develop and refine the decision aid. We then tested the decision aid in an uncontrolled trial. The primary outcome was the proportion of patients who were prepared to make an individualized decision, defined *a priori *as having adequate knowledge (10/15 questions correct) and clear values (25 or less on values clarity subscale of decisional conflict scale). Secondary outcomes included overall score on the decisional conflict scale, and preferences for undergoing screening.

**Results:**

We enrolled 46 adults in the trial. The decision aid increased the proportion of participants with adequate knowledge from 4% to 52% (p < 0.01) and the proportion prepared to make an individualized decision from 4% to 41% (p < 0.01). The proportion that preferred to undergo CRC screening decreased from 67% to 61% (p = 0. 76); 7 participants (15%) changed screening preference (5 against screening, 2 in favor of screening)

**Conclusion:**

In an uncontrolled trial, the elderly participants appeared better prepared to make an individualized decision about whether or not to undergo CRC screening after using the decision aid.

## Background

Colorectal cancer (CRC) screening is effective in decreasing disease-specific mortality in adults 50- 75 [1-3] but evidence about the effectiveness of CRC screening is limited for adults age 75 and older [4-7]. Extrapolating from trials in younger populations, it appears that factors, such as age and health status (and their effects on life expectancy) are important for determining whether older individuals could realize net benefit from CRC screening. The U.S. Preventive Services Task Force recommended in 2008 that persons aged 75 years and older not undergo *routine *CRC screening, indicating that the potential to benefit from screening should be considered at an individual level [8]. Similarly other expert groups, including the American Cancer Society, and the American Geriatrics Society, recommend that decisions about whether or not to undergo cancer screening in older adults are individualized based on the expectation of benefit, burden and potential harms of screening, and patient preference [9,10]. Taken together, guidelines suggest that decision making about whether or not to undergo CRC screening be individualized based on both: 1) consideration of the patients' health status and likely longevity; and 2) patient preferences about screening once they are informed about the potential benefits and harms.

Despite these recommendations evidence suggests that decision making for CRC screening in older adults could be improved [11]. Ideally, individualized decision making would promote screening in those who are healthy and most likely to benefit, discourage screening in those with multiple co-morbid conditions who are most likely to be harmed from screening, and educate patients so that their preferences about whether or not to undergo screening are informed [6]. However, observational data indicate no consistent association between screening test completion and health status [11-15]. Furthermore, older adults may be inadequately informed about the potential benefits and harms of cancer screening [16,17], and the elderly may not understand the effect of competing causes of mortality on the net benefit from undergoing screening [18]. Effective interventions to assure that patients are appropriately informed and have considered their personal preferences during colorectal cancer screening decision making are needed to ensure patients receive high-quality, guideline-concordant care.

One potential method for improving decision making is through the use of patient decision aids. In randomized controlled trials, use of decision aids compared to usual care has been shown to increase knowledge, decrease decisional conflict, reduce the proportion of people who are undecided, and increase the proportion who participate actively in decision making [19]. However, to our knowledge only one decision aid has been designed to promote individualized decision making in older people, that being for mammography in older women [20].

Effective decision aids have been developed and tested to assist colorectal cancer screening decisions in middle-aged adults [21,22]. These decision aids addressed decisions regarding CRC screening test choice. They did not target older adults for whom the decision of whether to undergo screening rests on how likely screening is to benefit an individual. Efforts to educate older adults about the efficacy of screening have been successful in increasing knowledge in adults age 65 and older [23-25], however, these studies were limited because the educational information provided did not consider health status. Additionally, neither study explicitly addressed patients' preferences by assessing their feelings about specific attributes of the screening decision.

To begin to address these gaps in the existing research, we sought to develop a targeted decision aid for adults age 75 and older designed to promote individualized decision making. Our goals for this study were to develop an acceptable, understandable decision aid and determine whether the decision aid could prepare older adults for individualized decision making. We first describe the steps we took to develop and formatively test and refine the content of the decision aid. Then, we report the results of an uncontrolled trial on several decision making outcomes among participants age 75 and older who used the decision aid. Because individualized decision making outcomes and processes could be influenced by participant characteristics, we also conducted exploratory analysis to assess whether these decision making outcomes were associated with participant characteristics, such as literacy, patient demographics, and health state.

## Methods

The study was conducted in two phases, a developmental stage and a testing stage. For the developmental phase we evaluated the decision aid content using cognitive interviewing techniques. For the testing phase we determined the effect of the decision aid on several decision making outcomes, using a pre-post test design.

### Development Phase

#### Recruitment and Eligibility

For the development phase, we recruited a convenience sample of participants from a local senior center. Older adults were eligible if they were age 75 and older and could read and speak English. We used two methods to recruit participants. We approached seniors at the center in person and invited them to participate. If they agreed and were eligible, the senior center provided a private room in which the participants and the research assistant could interact. In addition, we contacted elders who were participating in a pharmacist program of medication management. These elders qualified for the medication management program if they were homebound and on multiple medications. During one of her visits to the homes of elders participating in the medical management program, the pharmacist asked for their permission for us to contact them. If permission was granted, the pharmacist provided contact information to our research assistant who called potential participants at their homes. If they chose to participate, our research assistant (RA) arranged an appointment with them either in their home or at the senior center. For this phase of the study, there were 15 participants who were interviewed.

#### Decision Aid Development

We based the content of the decision aid on several conceptual frameworks. Walter and Convinsky proprosed a framework of individualized decision making for elders facing cancer screening decisions [6]. They proposed that the decision about cancer screening in the elderly depends on an assessment of the potential net benefit from undergoing screening and patient preference. Underlying this individualized decision making framework for the elderly is the more general concept of informed decision making. For people to make an informed decision they must be aware of the risk, benefits, alternative, and uncertainties inherent in the medical decision [26-28] to develop tools to assist patients so that they can make informed medical decisions consistent with their personal values. Based on the Ottawa framework and internal standards for decision aid development [29] we developed two components for the decision aid, an educational component and a values clarification component.

#### Educational Content

In the first phase, we developed and tested key messages which would facilitate individualized decision making for elders. From the existing literature, we identified information about the risks of CRC compared to other common causes of death in older adults (stroke and coronary heart disease) and information about the potential benefits of CRC in the general population, as age specific information was not available at the time. From in-depth interviews with adults age 75 and older [18] we identified a lack of knowledge about both the delayed benefit from screening and the need to make an individualized decision about CRC screening. From these data, we developed 5 key messages for formative testing:

1) There is a lack of direct research evidence supporting screening for those ages 75 and older; therefore, The American Cancer Society recommends that adults age 75 and older decide whether or not to get screened for colon cancer.

2) The risk of dying from CRC increases with age.

3) The risk of dying from of other common diseases also increases with age.

4) The importance of considering competing causes of mortality when determining whether CRC could be beneficial for older adults.

5) Colon cancer is relatively slow growing; people must be expected to live 5 to 10 years to have their life saved from colon cancer screening.

Using these messages, we performed the first round of cognitive interviews to refine the content of the elder-specific-decision aid messages. We tested whether participants: 1) could understand the information in each message; 2) found the information acceptable (and not offensive), and 3) thought the information was important to their decision about colorectal cancer screening. The messages were modified in an iterative fashion and cognitive interviews were continued until we reached saturation, that is we were not obtaining additional feedback. We completed a total of 15 cognitive interviews at this stage of development. The general concepts of the decision aid were understood by participants, thought to be important to decisions about CRC screening, and not found to be offensive to respondents.

With these messages as the core content, we then developed a paper version of the decision aid. The five key messages were incorporated into the paper based decision aid explaining why individualized decision making is important (Table 1). In addition to this section, we developed an introductory page outlining who should use this decision aid; a section of educational information briefly describing two screening tests (colonoscopy and fecal occult blood testing). In this section we explained that although fecal occult blood testing was an option, if the cards were positive, then colonoscopy would be needed to rule out the possibility of CRC. Consequently, the decision about whether or not to undergo CRC screening should be made by considering of the risks and benefits of colonoscopy. We also included a brief description of treatment options if CRC is found. We developed graphics demonstrating risk information including the potential benefits of CRC screening, risks of having serious complications from colonoscopy (bleeding, perforation, and death); and graphics demonstrating the balance of the risks and benefits for people in good, fair, and poor health states. Information about the risk of dying from CRC compared to stroke and heart disease was targeted to the participants' age and gender. The targeted information was presented in 5 year age increments 75 to 79, 80 to 84, and 85 to 90. Stroke and heart disease were chosen because they are leading causes of mortality in the elderly. This information was based on risks reported by Schwartz and colleagues describing competing causes of mortality [30].

**Table 1:** Summary of the Educational Content of the Decision Aid by Section.

Title of Section	Summary of content
Introduction	• American Cancer Society (ACS) recommends individualized decision making for older adults age 75 and over. • This decision aid will help you think about whether colorectal cancer screening is the right choice for you.

Information about Colon Cancer Screening	• Colorectal cancer screening tests look for colon cancer before you have symptoms.

Two Main Types of Tests that Screen for Colon Cancer	• Colonoscopy is a procedure that requires preparation and occurs at the doctor's office. • Stool cards can be done at home and returned to the doctor's office. Those with cards positive for blood will need to have a colonoscopy.

Treatments People Undergo if Colon Cancer is Found	• Most people with invasive colon cancer will need surgery. • Some people may need chemotherapy after surgery.

Colon Cancer Screening Recommendations Are Different for Older Adults	• As adults get older they are more likely to encounter numerous health problems that could affect their life expectancy. • We are not sure whether screening is beneficial for those 75 years and older. • That is why the ACS recommends older adults decide about colorectal cancer screening for themselves.

Why do Older Adults Need to Decide for Themselves about Colon Cancer Screening?	• The chances of getting a serious illness go up with increased age. Older adults are also more likely to develop colon cancer. Life expectancies for older adults vary with the number of serious health problems. • In most cases colon cancer grows slowly. If someone develops colon cancer today he may not have any problems for 5-10 years. • Colon cancer screening will not help all older people. A person's life expectancy can be influenced by their current health condition. • Older adults must deal with competing causes of death. Other health problems may lead to death before colon cancer. • There is uncertainty about who will benefit. No one can know how long any individual will live.

Magnitude of potential benefit from colon cancer screening	• One life is extended for every 1000 people who are screened.

**Figure 1:**
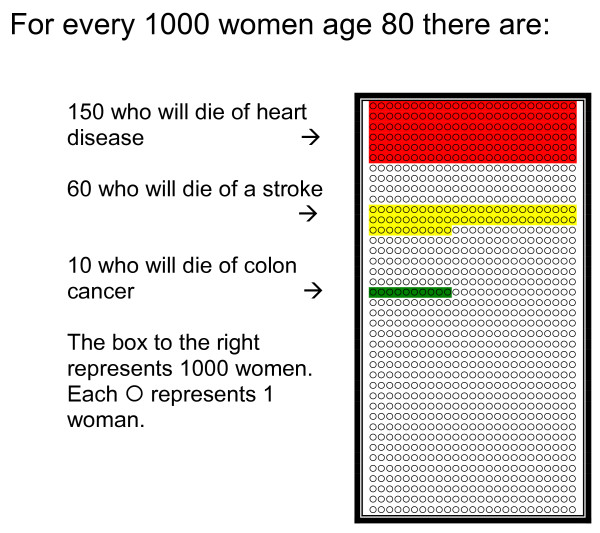
**Risk of Dying from Colon Cancer Compared to Other Common Diseases in the next 10 years**. This figure shows how colon cancer deaths compare to heart disease and stroke related deaths. There were 6 versions of this figure available because the decision aid was targeted to the participants' age/gender. This particular figure is for females age 80 and above. Each ◯ represented 1 person out of 1000 people in the figure.

**Figure 2:**
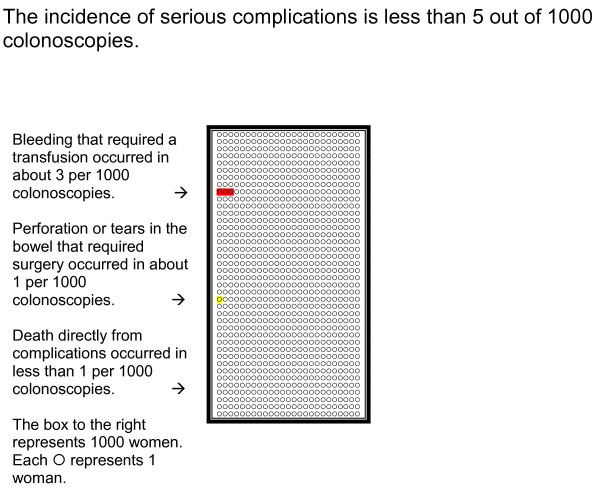
**Risks of Having Serious Complications from Colonoscopy within the first 30 days**. This figure shows information about the risks for a complication within the first 30 days after a colonoscopy. Each ◯ represented 1 person out of 1000 people in the figure.

Risks to Consider in Making Your Decision about Colon Cancer Screening	• Pictograph (Figure 1) compares the risk of dying from heart disease, stroke or colon cancer over 10 years. • Pictograph (Figure 2) compares the risk of having a complication (bleeding, perforation or death) after the first 30 days of a colonoscopy.

**Figure 3:**
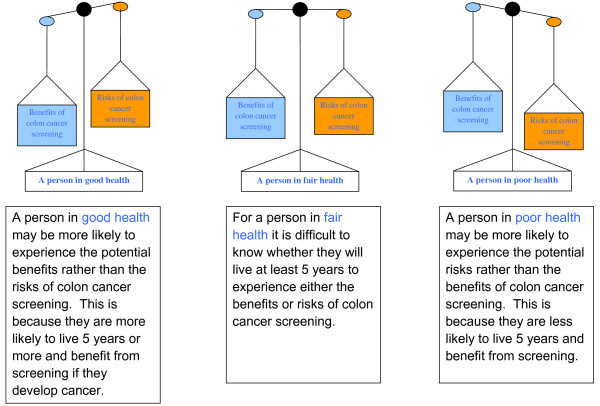
**Balancing the Benefits and Risks of a Colonoscopy**. Three different balance scales were shown to represent how CRC screening may or may not be beneficial for someone in 3 states of health (good, fair or poor). The scales showed how benefits or risks could outweigh each other or balance out depending on health state. A brief explanation for each scale was also provided underneath each picture.

Balancing the Benefits and Risks of Colonoscopy in People age 75 and Older	• Figure 3 compares how a person's health can influence the balance between the benefits and risks of colon cancer screening.

The table is divided into 2 columns. The first column lists the title of each section in the decision aid. The second column is a summary of the content in each section. Full content is available at http://www.shareddecisionmaking.org/Site/Female%20Age%2080.pdf

#### Values Clarification Content

As we developed and tested the educational component of the paper-based decision aid, we also developed and tested the content and process for a values clarification exercise. As there is no standard values clarification process, we based our process on an exercise developed for a prostate cancer screening decision aid by one of our co-authors. (Golin, CE, personal communication, April 2007). Our goal was to have a process that reflected the participants summed responses to the statements, so that participants could compare the results of the score from the values clarification exercise in the decision aid with their stated screening preference. We identified nine constructs related to decisions about colorectal cancer screening in older adults that could vary depending on older adults' values and could be important as they make decisions about cancer screening (Table 2). These constructs were identified as important for decision making during interviews with older adults [18]. Two cards were developed for each construct: one in favor of screening and one not. The participants reviewed the nine pairs of color-coded cards one at a time, and for each pair, chose which card best represented how they felt about that screening-related construct. The blue-colored cards supported screening, while the yellow colored cards were against further screening. Each choice was scored as +1 in favor of screening or -1 against screening. The sum of the choices was used to generate a score from -9 (nine choices against screening) to +9 (nine choices in favor of screening). At the end of the exercise the RA summarized the number of cards in favor (blue) and the number against (yellow). For example, "It looks like you've selected 7 blue cards and 2 yellow cards. The blue cards represent statements you might say if colon cancer screening was something you would want to do. The yellow cards represent statements you might say if colon cancer screening was something you weren't interested in doing. Since you selected more blue than yellow cards it looks like you are leaning towards colon cancer screening. Would you agree with that?" If they did not agree, the participant was encouraged to explain why they did not.

**Table 2:** Statements Used in the Value Clarification Exercise.

Construct	For CRC Screening	Against CRC Screening
Risk of Cancer	It is important to me to get screened for colon cancer even though the risk of getting colon cancer is small.	It is not important for me to get screened for colon cancer because the risk of getting colon cancer is small.

Functional Status	I understand that the prep and colonoscopy can be difficult but I don't think it would bother me that much.	I understand that the prep and colonoscopy can be difficult and I think it would bother me.

Priority	Based on my present condition, colon cancer screening is important compared with other health concerns.	Based on my present condition, colon cancer screening is not important compared with other health concerns.

Other Screening Decisions	I like to prevent health problems before I have symptoms.	I don't like to look for health symptoms that aren't causing me problems.

Treatment	I would want surgery if colon cancer was found even though it may not extend my life.	I would not want surgery if colon cancer was found even if there was a chance it could extend my life.

Worry	Getting colon cancer screening would give me peace of mind.	Getting colon cancer screening would not give me peace of mind.

Knowing I Have Cancer	I would want to know if I have cancer even if the cancer would not cause me any problems.	I do not want to know if I have cancer if the cancer would not cause me problems.

Complications From Screening	I am willing to take the risk of having a complication in order to have a chance to benefit from colon cancer screening.	I am not willing to take the risk of having a complication in order to have a chance to benefit from colon cancer screening.

Uncertainty	It is important for me to be screened for colon cancer even though it is uncertain whether or not it will prolong my life.	It is not important for me to be screened for colon cancer because it is uncertain whether or not it will prolong my life.

The table is divided into 3 columns. The first column lists the construct covered by each card. The second column displays a statement that is in favor of screening. The third column displays a statement that is against tscreening

#### Final Content of the Decision Aid

We performed another round of cognitive testing using the complete decision aid which included the informational component and the values clarification exercise. For this round of testing, the participants read each page of the decision aid and reflected back their understanding and impressions of the content. For each page, we evaluated the text, layout, graphics, and figures (Figures 1, 2, and 3). They also completed the values clarification exercise and provided verbal feedback to the RA about the process and the content. The process was iterative. We completed seven cognitive interviews testing the complete decision aid. Few changes were necessary. Some wording was changed to enhance understanding. The only substantive feedback was that the participants wanted us to better clarify that the outcomes from the risk tables were averages, and on an individual level, it is impossible to know who will benefit from screening. The final content of the educational component is available at http://www.shareddecisionmaking.org/Site/Female%20Age%2080.pdf.

### Testing Phase: Uncontrolled Trial

#### Recruitment and Eligibility

We recruited patients for the uncontrolled trial using the same methods described above. We recruited only those who had not participated in the formative testing. For the uncontrolled trial additional measures were obtained. The RA administered the Short Test of Functional Health Literacy in Adults [31], the Four Year Mortality Index [32], and a check list of medical conditions based on the Charlson Co-morbidity Index [33]. To exclude older adults with life expectancies of less than two years who would be unlikely benefit from screening, we planned to exclude people who reported end stage renal disease on dialysis, all oxygen dependent conditions, severe congestive heart failure, or terminal cancer but none that we recruited had these severe conditions. We also excluded people with a self-reported history of colon cancer. We screened potential participants for dementia using the Callahan's six item screen, a validated instrument[34], and excluded those with positive results.

#### Measures and Procedures

**Figure 4:**
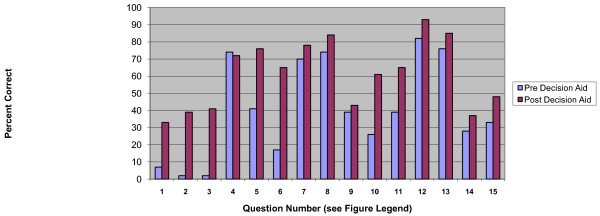
**Percent Correct for Knowledge Questions Before and After Decision Aid**. The percentage of correct answers on the 15-item questionnaire given Pre Decision Aid and Post Decision Aid. Participants responded to the following True/False questions: 1. No direct evidence supports screening. (T) 2. ACS recommends screening all adults. (F) 3. People in poor health are NOT likely to benefit. (T) 4. Longer a person lives the more likely they are to benefit. (T) 5. Risk of dying from heart disease is greater than dying from CRC. (T) 6. People need to live at least 5 years to benefit from screening. (T) 7. CRC screening is a choice for people ≥ 75. (T) 8. Tests look for colon cancer before symptoms. (T) 9. FOBT uses a lighted tube to check for CRC. (F) 10. CRC is the kind of cancer that grows quickly. (F) 11. Positive FOBT cards require a colonoscopy. (T) 12. During a colonoscopy polyps can be removed. (T) 13. Life expectancy is influenced by current health conditions. (T) 14. Not all people with CRC will need surgery. (T) 15. Bleeding and perforations are NOT complications of colonoscopy. (F)

After collecting the baseline information, the participant completed the self-administered pre-intervention questionnaire. This questionnaire included questions about participants' demographic characteristics, whether they had ever been screened for CRC, and whether or not they preferred to get CRC screening in the future. It also included the previously well-validated 16-item decisional conflict scale which includes the following subscales: informed, values clarity, support, uncertainty, and effective decision [35]. It also included self-reported health status, using a single question [36]. We assessed knowledge of colon cancer and CRC screening with a 15-item questionnaire drawn from previous questionnaires and our formative work (Figure 4). The knowledge questions were designed to determine basic knowledge about testing options and the key messages important for older adults presented in the decision aid. Upon completion of the pre-questionnaire, the participants read the decision aid booklet and participated in the values clarification exercise with the RA described above. After this exercise they completed a second, post-intervention questionnaire.

#### Outcomes for the Uncontrolled Trial

Our primary outcome was defined as the proportion of patients who were prepared to make an individualized decision of whether or not to undergo CRC screening. This outcome was based on the informed decision making model in which patients are adequately informed about the risks and benefits of screening and have considered their personal values about the decision [26]. Mathieu and colleagues developed this combined measure using knowledge and clear values for mammography screening in the elderly [20]. For our study, we defined *a priori *67% (10/15 questions correct) as adequate knowledge and clear values as 25 or less on values clarity subscale of decisional conflict scale because this cut point represents clear values. Secondary outcomes included the individual's knowledge and the clear values subscale, overall score on the decisional conflict scale, and whether or not they preferred to undergo screening. We also compared the results of the card-sorting values clarification exercise verbally with participants to determine agreement with their stated preference.

We performed exploratory analyses to test whether our primary and secondary outcomes varied according to some key co-variables that could have an effect on either decision making outcomes or screening preference, including literacy, health state, 4-year mortality index, number of chronic conditions, previous CRC screening, and demographic characteristics, such as age, gender, race, education, and income.

#### Perceptions of the Decision Aid

In addition, we asked questions to determine participants' perceptions of the decision aid and to identify areas that may need to be revised. We asked them to rate each of the six sections of the decision aid and the values clarification exercise using a 4 point Likert scale from poor to excellent. Finally, we asked about the length, the amount of information and whether the decision aid was useful.

#### Data analysis

First, we calculated frequencies for categorical data. To test the differences in responses to questions before and after the decision aid, we used McNemar's test for categorical measures and paired t-tests for continuous measures. To assess associations between participant characteristics and post-decision aid outcomes (knowledge, clear values, prepared to make an informed decision, and screening intent), we used Pearson's chi-square or Fisher's exact tests for categorical data and t-tests for continuous data.

#### Human Subjects

The study was approved by the Institutional Review Board at the University of North Carolina at Chapel Hill. Participants received $25 for their participation.

## Results

We recruited a convenience sample of 49 participants for the uncontrolled trial. Subsequently, one participant was excluded because she failed the cognitive screener, one was excluded because he reported a history of colon cancer after finishing the study, and one was not able to complete the literacy measure and was excluded. Among the remaining 46, three were legally blind and had the questions and decision aid read to them. Because of their inability to see, they were not able to participate in value clarification exercise. Our participants were primarily women (85%), most were white (72%), and 59% had completed at least some college (Table 3). Participants had a wide range of literacy levels, self reported health status, 4 year risk of mortality, and number of co-morbidities. Over half (65%) reported previous CRC screening.

**Table 3:** Participant Characteristics n = 46.

Mean age (range)	83 (75-95)
	N (%)

Gender	

Female	39 (85)

	

Race	

White	33 (72)

Black	11 (24)

Other	2 (4)

	

Education	

High school graduate or less	19 (41)

Some college or more	27 (59)

	

Previous CRC Screening	30 (65)

	

Literacy*	

Adequate	28 (64)

Marginal	5 (11)

Inadequate	11 (25)

	

Number of co-morbidities	

0-2	5 (11%)

3-7	27 (59%)

8+	14 (30%)

	

Self Reported Health Status	

Excellent/very good/good	20 (43%)

Good	17 (37%)

Fair/poor	9 (20%)

	

Four year mortality index	

< 4% risk	5 (11)

15% risk	18 (39)

42% risk	17 (37)

64% risk	6 (13)

Forty-six people participated in the study but 3 individuals were unable to complete the literacy assessment due to blindness

* N = 43, as 3 could not complete literacy assessment due to blindness

### Uncontrolled Trial Outcomes

#### Primary Outcome: Proportion prepared to make an individualized decision

Our *a priori *criteria for classifying participants who were prepared to make an individualized decision included 1) adequate knowledge defined as 67% of the true/false questions answered correctly (10 of 15 questions correct) *and *clear values defined as 25 or less on values clarity subscale of decisional conflict scale. Using these two thresholds, 4% were prepared to make an individualized decision before the decision aid and after using the decision aid, 41% fulfilled the criterion (p < 0.01).

#### Secondary Outcomes

The decision aid increased overall knowledge of colon cancer screening. At baseline 4% of the respondents reached the threshold for adequate knowledge by responding to 10 of the 15 true/false knowledge questions correctly. After exposure to the decision aid 52% of the respondents reached this knowledge threshold (p < 0.01). For five of the knowledge questions 70% or more of respondents responded correctly to the questions at baseline: 1) The longer a person lives the more likely they are to benefit; 2) Screening is a choice; 3) CRC screening tests look for cancer before they have symptoms; 4) During a colonoscopy polyps can be removed; and 5) Life expectancy is influenced by current health status (Figure 4). For these questions the increase in the proportion of people answering correctly after the decision aid was modest. For seven of the knowledge questions the proportion of participants who responded correctly increased by 25% or more after using the decision aid. These included the following constructs: 1) No direct evidence supports screening for adults ages 75 and older; 2) ACS recommendations for elderly 3) People in poor health are not likely to benefit from colon cancer screening 4) Risk of dying from heart disease is greater than dying from colon cancer 5) People need to live five years to benefit from screening 6) Growth rate of CRC 7) Positive FOBT cards require colonoscopy.

The proportion of respondents having clear values increased after viewing the decision aid, but the change was not statistically significant. (28 of 46 (61%) before vs. 32 of 46 (70%) after), Ten participants changed their clear value categories, 7 were unclear before the using the decision aid and became clear after using it while 3 were clear before the decision aid and unclear after its use (p = 0.27). Evaluating the full decisional conflict scale demonstrated a decrease in decisional conflict score after using the decision aid (mean score 34 vs 28 p < 0.01).

### Screening Preference

When we asked participants prior to the decision aid, 31 (67%), participants indicated that they preferred to undergo screening and after the using the decision aid 28 (61%) preferred to do so. Seven participants (15%) changed their screening intent after using the decision aid, with 5 deciding against screening after the decision aid and 2 changing in favor of screening. (p = 0.76).

### Values Clarification Exercise

**Figure 5:**
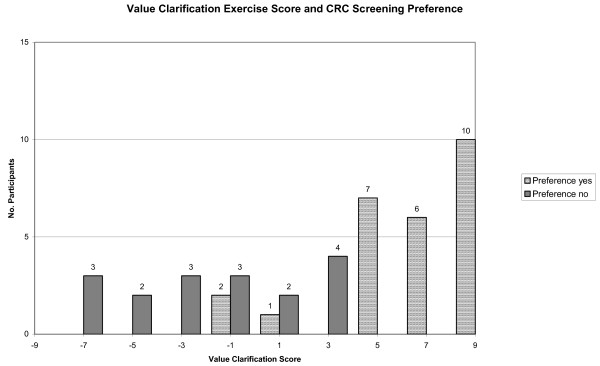
**Values Clarification Exercise Score and CRC Screening Preference**. Values clarification scores were summed according to cards the participant chose. Scores could range from -9 to 9. Those with negative scores indicated a preference against screening while those with postive scores indicated a preference for screening. We compared each participant's stated screening preference with their values score.

Among the 43 participants who completed the values clarification exercise, 13 had negative scores when the 9 cards were summed, indicating that the majority of cards they chose were against screening (Figure 5). The remaining 30 participants had positive scores when their cards were summed. When we compared participants' screening preference to the results of the values clarification exercise, we found that all 23 participants with scores on the card-sorting exercise of +5 or greater preferred screening. All 8 with scores of -3 or lower preferred to not have screening. In the middle range of scores from -1 to +3, 3 were in favor of screening and 9 preferred not to be screened. When asked at the end of the values clarification exercise, 32 (74%) of 43 participants agreed that their score represented their current preference for screening.

### Participants' Characteristics Associated with Outcomes

When we explored associations between participant characteristics and our primary and secondary outcomes, we found some potentially important associations despite the small numbers (Table 4). After using the decision aid, those less than age 83 were more likely to be prepared to make an individualized decision than those age 83 or older (59% vs 25% p = 0.02). Participants who had previously undergone CRC screening were more likely to have clear values (80% vs 50% p = 0.03) and prefer to be screened (73% vs 38% p = 0.02) Participants with adequate literacy were more likely to have adequate post-decision aid knowledge than those with inadequate or marginal literacy (64% vs 31% p = 0.04) and were more likely to be prepared to make an individualized decision after decision aid use: (54% of those with adequate literacy met the criteria, vs. 19% of those with inadequate or marginal literacy p = 0.03) Similarly, those with excellent to very good self-reported health status were more likely to have adequate knowledge and to be prepared to make an individualized decision compared to those with good to poor health (70% vs 38% p = 0.034 and 65% vs 23% p = 0. 004 respectively). As 4 year mortality increased, participants were less like to reach the threshold for clear values (p < 0.01) or preparation for an individualized decision making (p = 0.02) and were less likely to intend to undergo screening (p = 0.04).

**Table 4:** Associations between Participant Characteristics and Outcomes.

Participant Characteristics	Percent Reaching Knowledge Threshold	Percent Reaching Clear Value Threshold	Percent Prepared to Make an Individualized Decision	Percent Reporting a Preference to be Screened
**Age**				

< 83 (n = 22)	64%	73%	59%	73%

≥ 83 (n = 24)	42%	67%	25%	50%

				

**Sex**				

Women (n = 39)	49%	67%	38%	59%

Men (n = 7)	71%	86%	57%	71%

				

**Race**				

White (n = 34)	59%	74%	47%	56%

African-American (n = 11)	36%	55%	27%	73%

Other (n = 1)	0%	100%	-	100%

				

**Education**				

High School graduate or less (n = 19)	47%	63%	32%	58%

Some College or more (n = 27)	56%	74%	48%	63%

				

**Previous CRC screening**				

Yes (n = 30)	57%	80%	50%	73%

No (n = 16)	44%	50% *	25%	38%*

				

**Literacy**				

Adequate (n = 28)	64%	79%	54%	54%

Marginal/Inadequate (n = 16)	31%*	56%	19%†	69%

				

**Self-reported health status**				

excellent/very good (n = 20)	70%	90%	65%	70%

good/fair/poor (n = 26)	38% *	54% †	23%†	54%

				

**Co-morbidities**				

0-2 (n = 5)	80%	60%	60%	40%

3-7 (n = 27)	59%	74%	48%	70%

8 or more (n = 14)	29%	64%	21%	50%

				

**4 year mortality index**				

< 4% (n = 5)	80%	100%	80%	100%

15% (n = 18)	61%	89%	56%	61%

42% (n = 17)	41%	59%	29%	65%

64% (n = 6)	33%	17%†	0% †	16% †

The table looks at the associations between participant characteristics and outcomes after decision aid use

*statistically significant at < 0.05 using Chi-square

†statistically significant at < 0.05 using Fisher's exact

### Participants' Perceptions of the Decision Aid

Forty-one (89%) of the participants reported that the decision aid was useful. All six of the sections of the decision aid and the values clarification exercise were highly rated with 38 to 43 of the participants ranking each of the sections good to excellent. Thirty-seven participants (81%) thought the amount of information was just right while 5 participants (11%) thought that there was too little information.

## Discussion

During the development phase of the decision aid, we found that participants understood the key messages, thought the information was important, and did not find the information offensive. During the testing phase, participants reported that the decision aid was useful and rated the each of the sections highly. The results of our uncontrolled trial demonstrated that participants were better prepared to make an individualized decision, our primary outcome, after using the decision aid than before its use. The improvement was due primarily to an improvement in knowledge scores after decision aid use, as little change in the value sub-scale of the decisional conflict scale was noted. Participants also had a decrease in overall decisional conflict after using the decision aid. Our exploratory analyses reveal some areas of future study. Specifically, participants with inadequate or marginal literacy did not demonstrate as great an improvement in knowledge than those with higher literacy. Similarly, those in poorer health state (either by self report or the 4 year mortality index) appeared to have less clear values about screening than those in better health.

Previous studies have evaluated educational materials about cancer screening in older adults. Wolf and colleagues found that older adults were able to comprehend information about the efficacy of CRC for people of all ages. However, specific information regarding differences in potential benefits due to advanced age or health state were not provided [23]. Resnick found that when older adults where encouraged to consider not only the advantages but also the disadvantages of health promotion activities like CRC screening, they may be less willing to undergo screening [24]. However, participants' understanding of the information was not formally evaluated, so it is unclear whether understanding the risks and benefits changed their screening intent.

In this study, we developed a decision aid which was composed of both an educational component and a values clarification exercise consistent with international standards of decision aid development [29]. Importantly, the information in the decision aid was targeted to participants' age and gender. Targeting is important for this decision because the likelihood of benefiting from screening depends on an individual's risk of competing causes of mortality which varies by age and gender.

Developing effective interventions to promote individualized decision making about cancer screening in the elderly is important to improve their decision making. Available data suggests suboptimal decision making is ongoing, despite expert groups' recommendations for individualized decisions in this age group. Recent data from the V.A. demonstrates that screening test completion among the healthiest veterans age 70 and older was similar to those in the poorest health; 47% compared to 41% respectively [11]. This could lead to net harm in those who are unlikely to benefit, as they are exposed to the risks of screening without the potential to benefit. Furthermore, those who could benefit from screening may not get the opportunity to complete screening tests if they are not offered screening tests because of their advanced age.

Interventions, such our decision aid, that target older adults and inform them about the risks and the benefits of CRC screening relevant to their situation and have individuals consider their personal preferences have the potential to improve the decision making process in clinical practice. This study is the first step to test this hypothesis. In this uncontrolled trial, improvement in knowledge of key facts needed to make an informed yet individualized decision about CRC in older adults was encouraging. However, the decision aid will need to be revised to more effectively reach inadequate and marginal literacy users as well as those in poorer health states.

The majority of the participants (61%) in this study reached the threshold for clear values before the decision aid with a 9% increase after using the decision aid. There are several potential reasons for this small change. First, the decision aid could have had little effect on helping participants clarify their values. Another possibility is that the participants were already clear about which course to take. A randomized controlled trial of a decision aid for breast cancer screening in women age 70 and older also had a high proportion of participants (> 80%) with clear values in both the intervention and control arms [20]. More than 80% in both groups reported that they would continue screening, suggesting perhaps that this population was clear that they wanted to continue screening. However, the investigators did not assess whether screening intent varied with health state or increasing age. Although the numbers are small, our data suggest that those who have shorter life expectancies, estimated by the 4 year mortality index, have less clear values. Being informed about the decreased benefit and increased risks of screening with increasing age could have created some cognitive dissonance about what they believe about CRC screening. In our future work, we plan to address this question in a larger sample.

The uncontrolled trial was designed as a pilot test of the decision aid we developed. Obviously, further testing is needed before definitive conclusions can be made. There are several limitations that deserve consideration in addition to those already mentioned. Although we used formative work to develop the content of the educational material and the values clarification exercise, additional information may be important to older adults making decisions about CRC screening that we have not captured. The study was limited by its uncontrolled design. The differences we saw could theoretically be due to temporal trends, but the time between the pre and post surveys was short, so we think this is unlikely. It is also possible that participants could have answered the follow up questions in socially desirable ways. This seems unlikely for the knowledge questions but could be possible for the values subscale questions. On the other hand, we demonstrated that we were able to recruit participants with advanced age and a wide distribution of health states, which will be key in future studies. This task can be challenging because often elderly patients who have numerous co-morbidities often opt out of participating in research studies. Finally, we explored potentially important associations between participant characteristics and outcomes. In doing so, we performed multiple comparisons; therefore, caution should be used in interpreting these preliminary results as some of the associations we found could have occurred by chance. Additional research is needed assess these associations with larger samples.

## Conclusions

In summary, we found that the targeted decision aid designed to assist the elderly in deciding whether or not to undergo colorectal cancer screening was acceptable and useful to participants. In an uncontrolled trial, the participants appeared better prepared to make an individualized decision about screening. Additional research is needed to determine whether the targeted decision aid would be useful in a clinical setting to prepare patients for discussions with medical providers.

## Authors' contributions

CL conceived and designed the study, drafted the manuscript, obtained funding, performed statistical analysis, and provided supervision and administrative support. CG helped conceive and design the study, performed analysis and interpretation of the data, provided critical revision of the manuscript for important intellectual content and conducted statistical analysis. CD acquired the data, provided critical revision of the manuscript for important intellectual content, offered administrative, technical, or material support. JG assisted in drafting the manuscript, provided critical revision of the manuscript for important intellectual content and performed the statistical analysis. JI acquired the data, provided critical revision of the manuscript for important intellectual content, and offered administrative, technical, or material support. LT helped conceive and design the study, provided critical revision of the manuscript for important intellectual content, conducted statistical analysis, and provided supervision. MP assisted in drafting the manuscript and statistical analysis. All authors read and approved the final transcript.

## Pre-publication history

The pre-publication history for this paper can be accessed here:

http://www.biomedcentral.com/1472-6947/10/54/prepub
